# The Utility and Reliability of Treadmill Tests for Monitoring Thoroughbreds in Training

**DOI:** 10.3390/ani16142187

**Published:** 2026-07-14

**Authors:** Allan James Davie, Rosalind Beavers, Josh Denham

**Affiliations:** 1 Australian Equine Racing and Research Centre, Ballina, NSW 2478, Australia; 2 Faculty of Health, Southern Cross University, Lismore, NSW 2480, Australia; rosalind.beavers@scu.edu.au; 3 Faculty of Health Sciences and Medicine, Bond Institute for Health and Sport, Bond University, Gold Coast, QLD 4226, Australia; jdenham@bond.edu.au

**Keywords:** thoroughbreds, treadmill, reliability, blood lactate, heart rate, testing protocols

## Abstract

**Simple Summary:**

The objective of this investigation was to test the reliability of five treadmill protocols that assess heart rate (HR) and blood lactate (Bla) responses during a standardized gallop in Thoroughbred horses. Standardized exercise tests form a vital part of assessing and monitoring fitness levels by measuring cardiovascular responses to controlled exercise loads. The investigation of specific treadmill training protocols, which are commonly used in Thoroughbred training in Australia, for their reliability (i.e., test–retest repeatability) and therefore potential effectiveness for monitoring fitness and the wellbeing of the horse, is an important area from both performance and animal welfare perspectives. When considering the design of an exercise test it was critical that the magnitude, stability and pattern of the physiological response to the test workloads were considered. Further, the test must demonstrate acceptable reliability, repeatability, practicality and useability, and ideally should be designed for easy integration into the normal training program. The results showed that gallop HR was a reliable variable with HRR reliability improving as recovery period extended from 60 s to 90 s. Therefore, the treadmill tests demonstrated acceptable reliability to measure both gallop HR and HHR responses, whereas BLa responses were less consistent.

**Abstract:**

This study evaluated the reliability of five treadmill protocols that assess heart rate (HR) and blood lactate (Bla) responses in Thoroughbred horses to determine their effectiveness in serving as potential tools for monitoring fitness and the wellbeing of racehorses. Of the five exercise tests, four consisted of repeated interval gallops at constant speeds (8 m/s and 10 m/s) and grade (6°) and one test of a continuous gallop at constant speeds (8 m/s) and grade (6°). Eight hundred and twenty-three gallops in total were performed by 64 horses. The test–retest reliability and absolute agreement between treadmill tests were assessed using Intraclass correlation coefficient (ICC) with a two-way mixed-effect model. Statistical significance was set at *p* < 0.05. Overall, the mean of the mean HR for each of the five test gallops was 203 ± 11.3 bpm. The mean HR ICC of the five tests of 0.756 indicates good reliability of the tests, as HRs during each gallop were consistent between tests. Protocol comparison showed that all protocols have good reliability with gallops 1 and 5 scoring highest (0.79: 0.81) and gallop 4 lowest at 0.621. The recovery mean HR ICC for 30 s, 60 s, 90 s and 120 s recovery were 0.421, 0.504, 0.595 and 0.571 respectively. Overall, the data suggests that gallop HR is the more reliable variable with recovery HRR reliability improving to an acceptable level at 60 s and 90 s recovery. Together, the treadmill tests demonstrated acceptable reliability to measure both gallop HR and HHR responses, whereas BLa responses were less consistent.

## 1. Introduction

Fitness monitoring is crucial to avoid overtraining, illness and injury during preparations for racing, with inappropriate training programs potentially leading to undertraining, overtraining and injury [1]. Therefore, the design and evaluation of a training protocol is crucial. Further, standardized exercise tests (SETs) are vital for assessing fitness levels by measuring cardiovascular responses to controlled exercise loads [1]. Traditional laboratory-based treadmill assessments, which utilize complex measurements of oxygen uptake (VO_2_), ventilation, blood gases, heart rate (HR) and blood lactate (BLa) responses to specific workloads have been previously used for assessment of fitness and performance [2,3,4,5]. Studies that have investigated the correlation of running ability and physiological variables in Thoroughbred racehorses reported that running speed in races correlated with treadmill BLa variables (VLa4) [2] and that a correlation existed between racing performance time form ratings and BLa and HR measures during a treadmill exercise test, with correlations of BLa (r ≈ −0.68) and HR (r ≈ −0.56) [3].

However, traditional laboratory fitness tests are costly and technically complicated, which makes them less accessible and impractical for some horse trainers. Further for most tests there is a lack of scientific evidence with respect to validation, repeatability and suitability for the specific sports discipline in which the horse performs [6]. Therefore, investigating specific treadmill training protocols, which are commonly used in Thoroughbred training in Australia, for their reliability (i.e., test–retest repeatability) and therefore potential effectiveness for monitoring fitness and the wellbeing of the horse, remains an important area from both performance and animal welfare perspectives.

When designing an exercise test, it is important that the magnitude, stability and pattern of the physiological response to the test workloads are considered. Further, the test must demonstrate acceptable reliability, repeatability, practicality and useability, and ideally should be designed for easy integration into the normal training regime.

The equine treadmill provides an ideal addition to Thoroughbred training as it can ensure appropriate training volumes by directly controlling session intensity and duration of work intervals. Conversely, these parameters can be difficult to control in traditional track-based training. Equine treadmill training has been used for decades, and it continues to gain popularity for maximizing performance in equine athletes. The development of training protocols that utilize the treadmill can potentially both increase the cardiac pre-load training concept and improve the capacity to utilize the oxygen (i.e., muscle mitochondria content and function) for ATP production. Both human and equine research has reported that training at intensities between 75 and 85% VO_2max_ stimulate changes in mitochondrial content, an essential aspect of improving VO_2max_ [7,8].

Considering the linear relationship between VO_2_ and speed, one can predict the speeds necessary to elicit the desired VO_2_. For a horse with a VO_2max_ of 156 mL.kg^−1^·min^−1^ [9] 75% of VO_2max_ occurs at speeds of approximately 8 m/s, with 85–90% of VO_2max_ at speeds equivalent to 10–12 m/s. These speeds typically lead to the development of muscle cross sectional area (hypertrophy) [7]. In total, utilizing the treadmill at speeds ranging from 8 to 12 m/s at 6° appears ideal to develop both the aerobic energy system and muscular strength. Since any test of aerobic fitness should be above the minimum effective dose to elicit physiological adaptations and that a treadmill speed of at least 8 m/s is sufficient to achieve a desirable stimulus (~75% VO_2max_), a treadmill test of at least 8 m/s may be appropriate for assessing cardiorespiratory fitness and for monitoring purposes.

Although laboratory-based tests generally achieve acceptable test–retest reliability, their practicality is often questionable, as they require specialized laboratory equipment, trained personnel to safely administer the tests plus they can be costly. Therefore, field testing has evolved such that it can be more practical for the trainer to implement it into the training schedule [10]. Additionally, the reliability testing is conducted over a short period of time, and reliability properties may be different over extended time periods [10]. Reliability refers to the independence of a test from measurement error or whether an assessment instrument gives the same results each time under identical setting and the same type of subject [11]. Test reliability, along with the smallest worthwhile change, enables one to determine whether the test scores genuinely reflect changes in performance, which can then be used for fitness monitoring.

For the development of a treadmill exercise test that was easy to administer, and which also could be used as part of an authentic gallop training session, it was considered that monitoring the responses to not only the gallop phase but additionally throughout the recovery period would be important and informative. The monitoring of heart rate recovery (HRR) has been shown to be an important tool in monitoring performance and fatigue in human athletes [12]. Recovery HR is affected by the activity of the autonomic nervous system [13] with parasympathetic reactivation and sympathetic withdrawal occurring immediately post exercise, with the parasympathetic reactivation tending to have a faster response time than the sympathetic withdrawal [14]. In contrast, Savin et al. (1982) [15] reported that sympathetic withdrawal affected HR mainly initially, while parasympathetic activation was predominant later in recovery.

In the human field, autonomic nervous system recovery has been shown to occur more rapidly in individuals with greater aerobic fitness. Therefore, an abnormal HRR pattern may indicate the potential for the emergence of overtraining syndrome or decreased performance. However, it has been reported that using HRR to predict physical performance based solely on poor HRR is not ideal [16]. In horses, Bitschnau et al. (2010) [17] described HHR as decreasing in a bi-exponential manner, with a faster initial and a slower secondary decline. Nonetheless, HRR use in the equine field has shown promise, with Wilson et al. (2019) [18] demonstrating that HRR measures following exercise predicted race position in National Hunt racehorses, which they suggested could serve as a useful guide for trainers.

The design of any protocol for Thoroughbred horses should also involve a workload duration and exercise intensity that elicits a steady state HR [6]. Further, the protocol and test equipment must be valid, reliable and sensitive [19]. Therefore, the objective of our study was to assess the reliability of five treadmill gallops that are commonly used in training, using HR and BLa measures. Such a reliable test has the potential to serve as a tool for monitoring the fitness and wellbeing of racehorses.

## 2. Materials and Methods

### 2.1. Animals

All horses (n = 64) were from the same professional training centre in Melbourne Victoria, Australia. Ages ranged from 3 to 8 years old. Testing occurred over a 24-month period with each horse’s testing being dependent on when they came into training. Four horses completed three tests; eleven horses completed two tests; with all remaining horses completing only a single test. The time between tests depended on their racing campaign. At the time of testing, all horses were race prepared and therefore had a high level of fitness. All horses were familiar with galloping on the high-speed treadmill.

The same testers (treadmill operators) were used for all testing, and the AERR app (https://www.epfa.au) was used for implementing the treadmill sessions and monitoring HR responses. This ensured that the same protocols were followed during each test. The AERR app contains the full test protocols and directs the user through the full protocol. At the end of the test, the data is transferred to a computer program for analysis. Further, to help standardize the conditions of the tests, the circumstances and training environment were kept as consistent as possible. Specifically, all tests were performed in the morning at the same venues for each horse.

### 2.2. Test Design

In the application of the research findings [1,7] of the ideal percentages of VO_2max_ for specific muscle adaptations, an average VO_2max_ of 156 mL.kg^−1^.min^−1^ [9] was used for calculations of speeds. From the graph of VO_2_ versus speed, a 75% VO_2max_ occurs at speeds of approximately 8 m/s, and 85–90% of VO_2max_ occurs at speeds equivalent to 10–12 m/s.

In the design of tests 1 to 4, the intensity of the work was maintained at a constant speed of 8 m/s and grade at 6°. The volume of work progresses from test 1 to 3 by increasing the number of gallops and the length of each gallop. The total volume of work for test 1 is three 1 min gallops (a total of 3 min work), for test 2 it is 2 × 2 min gallops (a total of 4 min work) and for test 3 it is 3 × 2 min gallops (a total of 6 min work). Gallop 4 is a continuous single 3 min gallop at 8 m/s. For gallop 5 the intensity is increased to 10 m/s; however, the duration was reduced back to 3 × 1 min gallops (a total of 3 min work). All test gallops formed part of each horse’s training program.

### 2.3. Test Protocol

The study consisted of five exercise tests performed on a motorized equine treadmill, each conducted as part of each horse’s training schedule (see Table 1). All horses completed a warm-up consisting of a 1 min walk at 0°, followed by a 5 min trot and 3 min canter at a 4° incline before each test. For horses that completed more than one test, testing was conducted on separate days with a minimum of three days between tests.

Polar heart rate monitors (Polar H10, Kempele, Finland) were used for monitoring HR. The electrodes were lubricated and placed under an elastic girth strap, on the near side, with one electrode placed superior to the other. Each horse’s HR was continuously recorded during the tests, with peak HRs recorded for each gallop and at 30 s intervals for 2 min into the recovery period. The HRR period commenced at the end of each gallop and was calculated by subtracting the peak HR at each 30 s interval from the peak HR for that gallop (peak HR–post HR).

Venous blood samples were collected by jugular venipuncture using 21-gauge needles into 5 mL vacutainers containing lithium heparin (Vacutainer Systems, Becton Dickinson (BD), Franklin Lakes, NJ, USA). All samples were collected two minutes post treadmill gallop for whole blood lactate measurement. Whole blood lactate concentration (mmol/L) was assayed using a Lactate Pro 2 analyzer (Arkray, Inc., Kyoto, Japan).

Test repeatability was assessed with each horse repeating the same test several times over a 24-month interval. 

### 2.4. Statistical Analysis

The SPSS software (SPSS version 13, Chicago, IL, USA) was used for statistical analyses. Intraclass correlation coefficient (ICC) was used to assess the reliability index in test–retest reliability analyses. A 2-way mixed-effect model with absolute agreement was used as it reflects the variation in measurements taken by an instrument on the same subject under the same conditions [20]. The coefficient of variation (CV) was used to assess the relative variability of each horse and gallop sessions HR. Graphs were created in GraphPad Prism 10.6.1 (892), GraphPad Software, LLC, Boston, MA, USA. Statistical significance was set at *p* < 0.05. 

## 3. Results

The 64 horses completed 823 gallops overall. Some horses completed more than one test, with four horses completing three tests and eleven horses completing two tests. The number of horses and number of tests completed for each horse for tests 1, 2, 3, 4 and 5 are presented in Table 2. HR and BLa data are expressed as mean ± SD. The mean of the mean HR’s for the five test gallops was 203 ± 11 bpm. The mean HR range for all five gallops was 201 to 207 bpm, and mean BLa ranged from 3.4 to 4.4 mmo/L (see Table 2). Both measures indicate only average and constant levels of cardiovascular and metabolic stress.

Intraclass correlation coefficient (ICC) and coefficient of variation (CV) of HR (i.e., mean gallop HR and HRR at 3 recovery timepoints) and BLa responses for each of the five tests are presented in Table 3. The mean HR ICC of 0.756 for the five tests indicates good reliability with heart rates during each gallop effort being consistent between tests. Protocol comparison showed that all protocols have good reliability with gallop 1 and 5 scoring highest, and gallop 4 lowest at 0.621 (Table 3).

**Figure 1:**
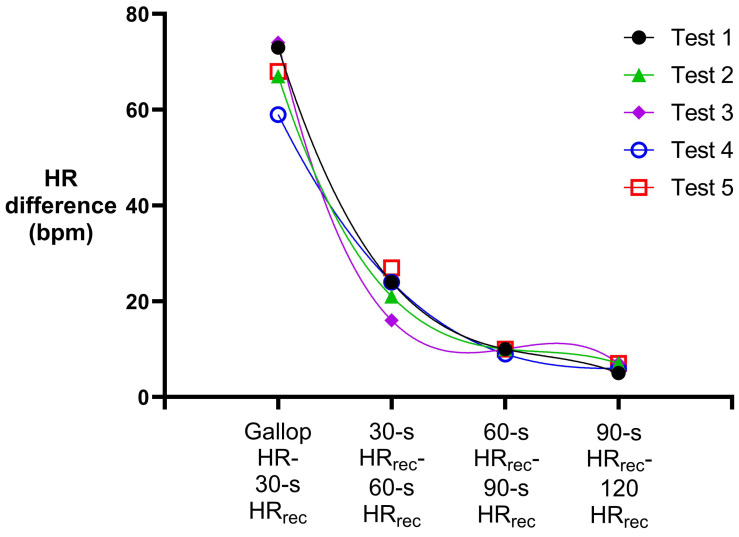
Graph of recovery HR data for treadmill tests 1, 2, 3, 4 and 5. The vertical axis shows the decrease in HR between each 30 s recovery interval. The lines represent the exponential graph of the data points for each test 30 s recovery interval.

Overall, the data suggests that gallop HR is a more reliable variable with HHR reliability improving after 60 s recovery, and that multiple sets of gallops in a test are slightly more reliable than the single stage test. BLa measures showed poor reliability with mean ICC of 0.415. This may be a result of sample variability with the range being only 2 to 4 mmol/L. The greatest rate of decline in HR was in the first 30 s with decreases ranging from 59 to 74 bpm. At 60 s the range had declined to 16 to 27 bpm with it dropping to 5 to 7 bpm by 120 s. This equates to a 36% drop in the first 30 s to only 6% drop from 90 s to 120 s of recovery (Figure 1).

In summary, the results demonstrate that the individual horse HR data is consistent and reliable, whereas the combined test gallop data is less reliable (or more variable) and therefore a less stable measurement. Practically speaking, using the test to monitor training is ideal due to the low CV; however, using the data to compare horses needs to be treated with more caution.

## 4. Discussion

The reliability of HR and BLa responses to five different treadmill exercise protocols was assessed using intraclass ICC and CV. Heart rate responses demonstrated good reliability overall (mean ICC = 0.756), indicating consistent responses during the gallops across the five tests. In contrast, BLa and HRR exhibited lower reliability, particularly at 30 s post exercise (ICC = 0.451), but improved progressively over time points, reaching moderate reliability at 60 s (ICC = 0.502), 90 s (ICC = 0.595), and 120 s (ICC = 0.571). This change in ICC from moderate towards good is indicative that the measures are more reproducible and stable as recovery progresses. This offers support to possibly assessing HRR at 90 s after exercise as the most appropriate timepoint. Across protocols, those that incorporated multiple sets (e.g., 3 × 1 × 8 m/s and 2 × 2 × 8 m/s) tended to demonstrate higher reliability compared to protocols with fewer sets, such as 1 × 3 × 8 m/s. Overall, gallop HR appears to be the most reliable physiological measure within these testing conditions, while early HHR and BLa are more variable in large groups of horses and should be interpreted with caution.

The CV assesses relative variability or how distributed the data is relative to the mean. A CV of less than 4% is considered low and demonstrates that any repeated tests on the same horse would only vary by about 4% of the mean, representing excellent repeatability. In this study a CV of 2.7% is considered low and provides strong support for using any of the repeated tests on the same horse. The mean CVs for the combined gallop and recovery periods were in the 5.8 to 9.3% range, and those for individual horse gallop and recovery periods were in the 2.7–6.2% range, indicating a much tighter clustering and more consistent (lower CVs) than the combined gallop data, which demonstrated more variability. In the between trial, the level of consistency between tests (gallops 1, 2, 3, 4 and 5) showed that the combined horse gallop HRs fluctuated more between trials (6.9 → 11.8 → 10.7%) than the individual horse HR data which is much more consistent (2.5 → 5.7 → 6.6%). The between trial supports the concept that any of the tests can consistently and accurately assess HR responses, particularly in individual horses, and are reliable for comparing HR changes over time. In contrast, BLa concentration showed poor reliability (mean ICC = 0.415), indicating a greater variability between trials. Blood lactate clearance reflects metabolic recovery and the body’s ability to buffer metabolic byproducts. While both are influenced by training status and endurance capacity, they are not interchangeable metrics. Despite the ICC and CV for lactate showing poor reliability, it is important to consider the dynamics of the calculations relative to the values measured. The mean BLa for the five gallops was 4.1 mmol/L, with range of 3.4 to 4.4 mmol/L. A change of only 1 to 2 mmol/L could represent a 25 to 50% variation, which would significantly impact on ICC and CV. Therefore, it is important that lactate measures should not be undervalued and could be considered in absolute terms or relative to lactate inflection point, such as VLa4 or individualized curve points.

In the equine field there are a variety of performance tests that have been used across different breeds for the assessment of poor performance and training adaptations. As discussed earlier, Harkins et al. (1993) [2] reported that running speed in races correlated with treadmill lactate variables (VLa4) and Evans et al. (1993) [3] reported both BLa (r ≈ −0.68) and HR correlated (r ≈ −0.56) with time form ratings. Seeherman and Morris (1990) [21] described the methodology necessary to perform a clinical exercise test and the expected variability in performance variables. The variability in all their measures was less than 5%, confirming that data obtained during a single exercise test can provide a reliable assessment of a horse’s metabolic capability. The current study adds support to these findings as all HR variability were less than 10% with some as low as 2.7%.

The use of heart rate measurements for predicting performance and monitoring training adaptations is based on a linear relationship between HR and workload during exercise. The derived parameters from these measurements have been used to predict endurance performance and in prescribing and monitoring training intensities [22]. All parameters of cardiac function, including HR, conduction, force of contraction and relaxation, reflect the net balance between an inhibitory parasympathetic influence and an excitatory sympathetic influence [23]. Cardiac parasympathetic reactivation following exercise is considered the principal determinant of the immediate fall in heart rate when exercise ceases, or intensity drops [24] with the parasympathetic reactivation having a faster response time than the sympathetic withdrawal [14]. However, Savin et al. (1982) [15] reported that sympathetic withdrawal affected HR initially, while parasympathetic activation was predominant later in recovery. The pattern for HHR is an initial sharp fall followed by a slow decrease [15] over many minutes [25,26]. In the human field it is well documented that HRR taken at select time points is lower in endurance-trained athletes [27] and has the potential to monitor changes in performance [12,22,24]. Hagberg et al. (1980) [28] showed that the adaptations to endurance exercise training enabled a more rapid rate of HRR. Although others have outlined that a faster HRR does not systematically predict better performance, they suggest that HRR after submaximal exercise may be more discriminant than HRR measures taken after maximal exercise [6]. Therefore, it seems that some key issues need to be addressed in the monitoring of HRR such as the mode of exercise (intermittent vs. continuous), duration and fitness level [24]. Further, assessing HRR after submaximal exercise may be more practical and the use of standardized exercise protocols before HRR is measured may be important to yield consistent results. Lamberts et al. (2010) [12] showed that measurement of HRR achieved its highest sensitivity to detect meaningful changes when exercise was at 86% to 94% of heart-rate maximum.

In the equine field it has been reported [18] that HRR following interval exercise could be used to predict race position in National Hunt races and that the speed of HRR to one minute after a high-speed training session predicted race performance; however, the effect was markedly reduced when the variation between horses was accounted for [29]. In the current study the absolute HR decrease from the gallop to 60 s recovery was in the range of 83–97 bpm, which is greater than the 68–70 bpm reported by Schrurs et al. (2014) [29]. This variation is expected considering that on the treadmill, the environment is more controlled with horses reduced to a walk at zero degrees by one minute post gallop. Therefore, even though the largest decrease occurs in the first 30 s post exercise the HR is more stable at the 90–120 s period as supported by the ICC which improved progressively over time. This large variability in the first 30 s recovery makes an understanding of the causes of this variability important to enable accurate application of meaningfulness to the data.

However, in reference to the practicality of using the treadmill results for assisting in monitoring and design of training programs, Bitschnau et al. (2010) [17] (in a study of Warmbloods) outlines some concerns. Although the results of exercise testing are often transferred into recommendations for daily training, caution is needed in that there are differences in metabolic response to track and treadmill exercise. They also point out that the transfer of results from treadmill testing to recommendations for actual training is not sufficiently established in horses and warrants further investigation.

To address the concerns of Bitschnau et al. (2010) [17] regarding the metabolic differences and practical application of a test, the data collected must be in a range commonly experienced by horses during training and is sufficient to provide a minimal stimulus to elicit the desired adaptations. In the current study the mean HR over the five gallop tests was 203 ± 11 bpm. Maximum heart rates in Thoroughbred horses have been reported in the range 210–240 (bpm) [30]. The average heart rate during the treadmill tests of 203 bpm represents approximately 80% of maximum heart rate (240 bpm). This intensity is within the recommended effort requirements (75–85% VO_2max_) necessary to elicit aerobic training adaptations. Early work by Sinha et al. (1993) [31] suggested that for major adaptations in skeletal muscle, an intensity of ~80% VO_2max_ may be more significant than the degree of exercise load, when exercise intensity is submaximal.

The reporting of good reliability overall for the physiological measures supports the use of any of the five gallops for assessing and monitoring horses in training. The CV of 2.7% is considered low and provides strong support for using any of the tests on the same horse to monitor changes in fitness and wellbeing.

The five treadmill tests are frequently used as part of training routines in Australian racing stables. The current research findings will provide confidence for trainers in using the tests not only as a part of their training but additionally to assess the training state and welfare of their horses. In terms of potential future applications of Thoroughbred training, these five gallops can be utilized as part of the implementation of the Pyramidal training concept. The Pyramidal training concept is popular in elite endurance and middle-distance athletes and is becoming more popular within the Thoroughbred industry [32]. In this training approach a large percentage of training is done at low–moderate intensity (70–80%) and 20–30% at moderate to high intensities [33]. Within this approach the importance of the prescribed training load is a key factor in eliciting physiological adaptations [34]. Training load is a combination of the intensity of the stimuli and the volume of training, plus the length of the recovery period between each work session. The load can be broken down into an external and internal component. The external load is the volume and intensity of the work whereas internal load is the psychophysiological responses that the load initiates to cope with the external load [34]. HR and BLa responses to the exercise training provide the best indices of internal load and are thus considered the most accurate measures of effort and can be used as a guide for setting the optimal training stimulus [35].

Accordingly, knowing the reliability of the five tests for measuring the internal training load (i.e., HR and BLa) and utilizing that information to control the training intensity (i.e., speed) and duration of training enables the trainer to achieve the desired training stimulus, thus representing a conceptually attractive training method. Further research could be directed at validation of these tests in both unfit and highly trained horses to establish its sensitivity to detect differences in performance. The sensitivity of the tests to identify changes in performance following standardized training programs should also be examined. Finally, since the treadmill tests rely on HR responses, factors that affect HR must be controlled (i.e., frightful stimuli, temperaments, etc.); otherwise, the test results will be confounded.

### Limitations

A limitation of the work is that it was conducted at one training centre, with all tests conducted in the morning. A further limitation could be that ambient temperature, humidity, and climate control during testing were not reported which may have a small effect on their physiological responses. However, despite these potential limitations, the scale of the data and the strength of the results support the validity of the findings. Despite the limitations, the manuscript involved a large cohort of race-fit thoroughbreds, objective measures of physiological stress and authentic gallops on an equine treadmill, all of which can add positively to the equine training world.

## 5. Conclusions

The data suggests that gallop HR is the more reliable variable with HRR reliability improving to an acceptable level at 60 s and 90 s recovery. Together, the treadmill tests demonstrated acceptable reliability to measure both gallop HR and HHR responses, whereas BLa responses were less consistent.

The results provide support for the application of these evidence-based training and testing methods for training analysis and potentially as a means for monitoring the fitness and wellbeing of racehorses.

## Figures and Tables

**Table 1:** The gallop protocols for each test, including the number of gallops, duration of gallop, speed (m/s) and treadmill grade (Deg). Column 1 lists each test. Column 2 is the number of gallops performed in each test. Column 3 lists the duration of each gallop (min). Column 4 outlines the speed of each gallop (m/s), while column 5 lists the treadmill grade used for each gallop. There was 5 min recovery between each gallop which included walking for 3 min at 0°, trot 1 min at 4° incline and canter 1 min at 4° incline. At the completion of each of the five tests (1–5), each horse walked at 0° for a 2 min active recovery.

Test	Number of Gallops	Gallop Duration (min)	Speed (m/s)	Grade (Deg)
1	3	1	8	6
2	2	2	8	6
3	3	2	8	6
4	1	3	8	6
5	3	1	10	6

**Table 2:** The average heart rate (HR) and blood lactate (BLa) responses during the five treadmill tests. Column 1 is the test number which represents the specific protocol (i.e., the different gallop protocols). Column 2 is the number of horses multiplied by the number of gallops completed by each horse (i.e., for each test protocol). The gallop HR is expressed in mean ± SD for all horses that completed the number of gallops. Columns 5–8 list the mean ± SD for HRR (bpm) at each of the recovery periods (Rec 30 s to Rec 120 s). Column 9 is the mean ± SD for BLa (mmol/L) for all horses that completed that test.

Test	Horses (n) × Number of Gallops (n)		Gallop HR	30 s Rec HR	60 s Rec HR	90 s Rec HR	120 s Rec HR	BLa
1	15 × 15	Mean	203	129	105	95	89	4
		SD	11.5	11.3	11.4	10.0	8.8	1.8
2	20 × 8	Mean	202	131	109	99	92	4.2
		SD	12.3	13.8	10.8	9.6	9.7	1.8
3	17 × 9	Mean	201	130	109	99	92	4.4
		SD	12.4	11.5	11.3	9.7	8.8	2.0
4	13 × 9	Mean	202	136	109	99	92	3.4
		SD	8.9	10.2	8.0	7.0	6.7	1.3
5	14 × 12	Mean	207	142	113	101	94	4.4
		SD	11.5	9.4	9.1	7.5	7.7	1.5
		Mean	203	134	109	99	92	4
		SD	11.3	11.2	10.1	8.7	8.3	1.7

**Table 3:** The reliability (ICC and CV) of HR responses to five treadmill tests and recovery periods, plus recovery BLa. The ‘test’ column represents the five treadmill tests (i.e., the different gallop protocols). Rows 1–7 list the mean ICC for the gallop, recovery periods and Bla for the five treadmill tests. Rows 9–15 list mean CV of HRs for test gallops, recovery periods and CV for Bla for the five treadmill tests. Rows 17–23 list mean CV of HRs for gallops, recovery periods and CV for Bla for individual horses for each test. Intraclass correlation coefficient (ICC) and coefficient of variation (CV) scores are presented as % for all tests.

Test	Gallop HR	Rec 30 s HR	Rec 60 s HR	Rec 90 s HR	Rec 120 s HR	BLa
1	0.797	0.568	0.61	0.655	0.605	0.49
2	0.784	0.666	0.594	0.726	0.694	0.45
3	0.768	0.362	0.492	0.516	0.485	0.46
4	0.621	0.152	0.399	0.477	0.437	0.22
5	0.809	0.355	0.424	0.602	0.633	0.46
Mean	0.756	0.451	0.502	0.595	0.571	0.415
SD	0.08	0.20	0.1	0.10	0.11	0.11
1	5.7	8.5	10.9	10.5	9.8	40.4
2	5.9	11.8	10.7	10.5	9.8	37.5
3	6.2	8.4	9.9	9.5	9.4	44.7
4	4.5	6.6	7.1	6.8	7.1	38
5	5.5	6.4	7.8	7.5	8.3	36.3
Mean	5.8	8.4	9.3	9	8.9	39.2
SD	0.89	2.2	1.7	1.7	1.2	3.6
1	2.5	5.7	6.6	5.9	5.9	21.9
2	2.9	6.3	5.9	5.1	5.1	24.9
3	2.9	6.4	7.1	6.6	6.4	22.4
4	2.7	5.5	5.6	5.2	5.4	30.1
5	2.3	5.4	5.9	4.6	4.7	23.3
Mean	2.7	5.9	6.2	5.5	5.5	24.5
SD	0.23	0.48	0.63	0.79	0.68	3.3

## Data Availability

All data is kept with the chief investigator (Allan James Davie) and can be made available by contacting him. However, there is a confidentiality issue with releasing individual horses’ results.
